# Integrative phosphoproteomics reveals kinase-mediated regulation of OCIAD1 and its roles in mitochondrial quality control

**DOI:** 10.3389/fbinf.2026.1770712

**Published:** 2026-07-23

**Authors:** Amal Fahma, Suhail Subair, Fathimathul Lubaba, Athira Perunelly Gopalakrishnan, Prathik Basthikoppa Shivamurthy, Rajesh Raju

**Affiliations:** Centre for Integrative Omics Data Science (CIODS), Yenepoya (Deemed to be University), Mangalore, Karnataka, India

**Keywords:** co-differential regulation, mitochondrial quality control, OCIAD1, phosphoproteomics, upstream kinases

## Abstract

**Background:**

The ovarian cancer immunoreactive antigen domain-containing protein 1 (OCIAD1) is a mitochondrial protein implicated in mitochondrial morphology, energy metabolism, and differentiation. Although understudied, recent studies position it as a critical player in carcinogenesis and neurodegenerative disorders, making it a potentially druggable node in cellular signaling networks. However, the phosphoregulatory networks and the upstream kinases governing OCIAD1 remain unknown.

**Methods:**

A large-scale literature mining and analysis of 177 phosphoproteomic datasets with differential expression of OCIAD1 was carried out to map its phosphoregulatory network. The predominant phosphosites were determined based on localization probability, detection frequency, and differential regulation. Multipronged computational approaches were employed to gather novel candidate kinases that may target OCIAD1 phosphosites. Co-differential phosphorylation analysis was conducted with other proteins, including interactors and candidate upstream kinases, to infer functional and regulatory associations.

**Results:**

The sites S108 and S123 emerged as predominant, together accounting for 70% of OCIAD1 phosphorylation. Co-differential phosphorylation analysis revealed associations with proteins involved in the cell cycle, DNA repair, autophagy, mitophagy, endocytosis, and apoptosis. Novel candidate kinases for OCIAD1 phosphosites were identified; notably, SRMS and YES1 emerged as potential upstream regulators of Y199. Furthermore, the phosphosites in the candidate kinases of sites, including PLK1 (T210), CDK13 (S383, S397), PRKD2 (S200), CIT (S1343), and RPS6KA3 (T577), showed strong positive co-differential regulation with OCIAD1 predominant sites, supporting their potential involvement as upstream kinases.

**Conclusion:**

This study presents the first systematic map of the OCIAD1 phosphoregulatory network and provides candidate upstream kinases that may contribute to its phosphorylation, which warrant further experimental validation. The strong co-differential regulation of proteins involved in autophagy, mitophagy, endocytosis, and neurodegenerative pathways, as well as of kinases that orchestrate these processes, suggests that OCIAD1 phosphoregulatory network maybe involved in mitochondrial quality control and mitochondria-associated neurodegeneration, establishing a foundation for therapeutic investigations targeting OCIAD1 signaling.

## 1 Introduction

The ovarian cancer immunoreactive antigen domain-containing protein 1 (OCIAD1) has recently emerged as a key modulator of mitochondrial morphology, energy metabolism, and differentiation (Praveen et al., 2020; Shetty et al., 2018). It is a poorly characterized inner mitochondrial membrane protein belonging to the OCIAD1 family and is also known as ASRIJ (Le Vasseur et al., 2021). OCIAD1 was first identified as an immunoreactive antigen through immune screening of an ovarian carcinoma cDNA expression library using ascites fluid from ovarian cancer patients (Luo et al., 2001). The protein localizes to endosomal compartments and mitochondria, regulating mitochondrial morphology and energy metabolism through its interactions with electron transport chain complex I (Shetty et al., 2018; Mukhopadhyay et al., 2003; Calvo et al., 2016). Notably, depletion of OCIAD1 results in elongated mitochondria and diminished mitochondrial dynamics, leading to aberrant progenitor differentiation (Praveen et al., 2020; Shetty et al., 2018). It modulates several signaling pathways, including JAK/STAT, Notch, and PI3K/AKT, influencing cell fate (Ra et al., 2021; Sinha et al., 2013). Asrij plays a critical role in sustaining pluripotency in mouse embryonic stem cells and in maintaining hematopoietic stem cells in *Drosophila*; its loss in either system results in abnormal cell differentiation (Sinha et al., 2013; Kulkarni et al., 2011). It is also involved in STAT3 and ERK phosphorylation and may act as a scaffold for the activation and interactions of STAT3 (Sinha et al., 2018; Hornbeck et al., 2012).

OCIAD1 is recognized to be a contributor to cancer progression, with its upregulation associated with increased cell migration, invasion, and metastasis in multiple cancers, including ovarian, thyroid, and pancreatic ductal adenocarcinoma (PDAC) (Ji et al., 2019; Yang et al., 2012; Sengupta et al., 2008; Wang et al., 2010). The elevated OCIAD1 levels improve integrin function and strengthen interactions with the extracellular matrix, promoting metastasis (Wang et al., 2010). In PDAC, overexpression of OCIAD1 promotes tumor cell migration and contributes to malignancy by suppressing ATM kinase activity. The reduced ATM function impairs DNA damage repair, thereby promoting genomic instability and tumor progression (Ji et al., 2019). Recent studies found the upregulation of OCIAD1 also has implications for neurodegenerative disorders, including Alzheimer’s (Dongre et al., 2025; Geertsma and Rousseaux, 2020; Li et al., 2020).

A study by Wang et al. suggests that the functions of OCIAD1 may be closely regulated by its phosphorylation status. Upon stimulation by signaling molecules such as lysophosphatidic acid (LPA), OCIAD1 undergoes serine phosphorylation, and this modification likely governs its activities and drug sensitivity (Wang et al., 2010). Protein phosphorylation is an important post-translational modification (PTM) that regulates various processes, including cell growth, differentiation, and aging through the coordinated actions of kinases and phosphatases (Ardito et al., 2017). It plays a key role in cell signaling by regulating protein–protein interactions, subcellular localization, and complex signaling networks (Nishi et al., 2011; Andjelković et al., 1997; Ardito et al., 2017; Rosen and Erlichman, 1975). Aberrant protein phosphorylation is a hallmark of various diseases, including cancer, and is associated with altered cell proliferation, migration, and metabolic regulation (Abramczyk et al., 2019; Ahmed et al., 2017).

Despite its emerging significance, particularly in carcinogenesis, drug resistance, and neurodegenerative disorders, very little is known about how OCIAD1 is regulated through phosphorylation. Understanding which kinases or phosphatases act on OCIAD1 and how its phosphorylation affects its function could reveal new therapeutic strategies. There are no studies to date that have identified particular kinases or phosphatases that modulate OCIAD1 phosphorylation. The capability of mass spectrometry (MS) to detect and measure a large number of proteins has revolutionized the fields of biology and medicine (Aebersold and Mann, 2003). It has also enabled the large-scale, high-throughput identification and quantification of phosphosites across the proteome (Yu and Veenstra, 2021). Its application in research is emerging due to the critical role of phospho-signaling in normal and pathological conditions (Kauko et al., 2015; Gerritsen and White, 2021). Thus, we aimed to build an integrative, data-centric co-differential phosphoregulatory network of OCIAD1’s phosphosites to better understand the functional aspects of OCIAD1 phosphorylation by integrating global mass spectrometry-based phosphoproteomic data and bioinformatic approaches.

## 2 Materials and methods

### 2.1 Compiling of phosphoproteomic datasets with OCIAD1 phosphosites

A PubMed search was carried out using the terms “phosphoproteomics” OR “phosphoproteome.” The studies on plants and review articles were excluded and only studies reporting mass spectrometry–based phosphoproteomic datasets were consideredThe articles comprised phosphoproteomics datasets generated under diverse experimental conditions and biological systems, including cell lines, tissues, and animal models. From these datasets, those derived from human cell lines were selected for further analysis. Through a multi-step manual curation, each retrieved study was screened by examining the associated supplementary phosphoproteomic datasets to identify those reporting OCIAD1 phosphosites. The curated datasets included OCIAD1 phosphopeptides generated through enzymatic digestion using LysC and/or trypsin, which are standard proteases used in phosphoproteomics workflows. The datasets obtained were categorized as qualitative profiling datasets (where test and control conditions are treated as distinct datasets) and quantitative differential datasets (experimental conditions compared with their respective controls).

### 2.2 Selection of high-confidence phosphosites and differential expression analysis

We prioritized phosphosites exhibiting a localization probability above 75% or an A-score above 13 (termed as class 1 phosphosites) to guarantee the reliability of data and omit false positives. The localization probability for a site is defined as the normalized sum of the probabilities in instances where the site is harboring a modification (Sharma et al., 2014). Increased localization probability, or A-score, assigned reliability in detecting phosphosites. Data filtering was based on a p-value cutoff of <0.05 to assess statistical significance. Additionally, phosphosites with a fold change ≥1.3 were considered upregulated, and those ≤0.76 were considered downregulated according to the commonly used threshold in several curated phosphoproteomic studies. Furthermore, datasets were categorized in accordance with the approaches used for the enrichment of phosphosites (serine, threonine, and tyrosine). An in-house mapping tool was employed to link each phosphosite in the datasets to its corresponding UniProt accession.

### 2.3 Peptide mapping and sequence coverage analysis of OCIAD1

To visualize peptide coverage and PTMs on serine, threonine, and tyrosine residues, of the OCIAD1 protein, we employed a peptide mapping approach using Class I phosphosites obtained from global human phosphoproteomic profiling datasets. Modified OCIAD1 peptides identified in these datasets were analyzed. Sequence coverage and phosphosites were visualized using the Sequence Coverage Visualizer (SCV) (Shao et al., 2023)**.**

### 2.4 Identification of predominant OCIAD1 phosphosites

The detected phosphosites were ranked according to their detection frequency across diverse experimental conditions. The top OCIAD1 phosphosites representing OCIAD1 phosphorylation in global phosphoproteomics (predominant OCIAD1 phosphosites) based on their differential frequencies across datasets were chosen to analyze their co-differential regulation with phosphosites in other proteins.

### 2.5 Identification of phosphosites in other proteins co-differentially regulated with predominant OCIAD1 phosphosites

To identify the protein phosphosites in other proteins co-differentially regulated along with OCIAD1 predominant phosphosites, they were categorized based on the coregulation patterns observed in distinct differential datasets (Raghu et al., 2025; Priyanka et al., 2024; Fahma et al., 2025). Those protein phosphosites that are upregulated in conjunction with OCIAD1 predominant phosphosites are termed UU (both OCIAD1 and protein phosphosites upregulated), while those that are downregulated along with OCIAD1 predominant phosphosites are termed DD (both downregulated). Integrating these coregulation patterns, we named those protein phosphosites exhibiting the exact directional change, along with OCIAD1 predominant phosphosites, as similarly (positively) co-differentially regulated (UUDD). Additionally, those protein phosphosites that are downregulated while OCIAD1 predominant phosphosites are upregulated are termed UD (OCIAD1 upregulated, protein phosphosites downregulated), and those that exhibit upregulation alongside OCIAD1 downregulation are categorized as DU (OCIAD1 downregulated, protein phosphosites upregulated). Collectively known as oppositely (negatively) co-differentially regulated protein phosphosites (UDDU).

### 2.6 Filtering strategies for identifying strong coregulation across phosphoproteomic datasets

Many protein phosphosites demonstrated distinct regulatory patterns throughout the datasets. Hence, to evaluate the tendency of the phosphosites in other proteins to similarly or oppositely co-differentially regulate with the OCIAD1 predominant phosphosites, we examined the frequency of instances where neither, either, or both the OCIAD1 predominant phosphosites or their specific protein phosphosites were co-differentially regulated. Subsequently, we calculated the p-value from a one-sided Fisher’s exact test (FET) according to a 2 × 2 contingency table. The protein phosphosites with p-value <0.05 in the UUDD and UDDU sections for the predominant phosphosites of OCIAD1 were regarded as statistically relevant for further studies. They were then screened based on the confidence ratio for similar or opposite co-differential regulation. The number of datasets with similar co-differential regulation/number of datasets with opposite co-differential regulation (UUDD/UDDU) was considered for similarly co-differentially regulated protein phosphosites, and the number of datasets with opposite co-differential regulation/number of datasets with similar co-differential regulation (UDDU/UUDD) for oppositely co-differentially regulated protein phosphosites. A ratio representing at least 10% of the number of differential datasets for the OCIAD1 predominant phosphosites was defined as high confidence.

To mitigate any bias arising from the inclusion of phosphoproteome datasets representing temporal stages of the same study or datasets derived from the same experimental conditions or stimulus, we applied specific cutoffs. For this, we generated experimental codes to categorise all experimental conditions with the same stimuli into a single experimental code. By subtracting the negative coregulation experimental code count from the positive coregulation experimental code, we then generated an experimental code confidence value for positive coregulation and *vice versa* for negative coregulation. Similarly, we calculated the number of PMIDs from which the phosphoproteomics data were derived and calculated a PMID confidence value for positive and negative coregulation. We then applied the experimental code confidence and PMID confidence cutoffs of ≥2 to these positive and negative coregulations. Those coregulated phosphosites that satisfy these filtering parameters were then considered as potentially significant expression coregulated phosphosites with predominant sites of OCIAD1. To further refine the analysis.

### 2.7 Extraction of candidate upstream kinases and co-differentially regulated binary interactors of OCIAD1

To investigate the phosphorylation-centric landscape of OCIAD1, we performed the following analyses: (a) Prediction of upstream kinases that may phosphorylate OCIAD1 based on multiple prediction tools such as NetworKIN [32] and Automatic Kinase-specific Interactions Detection (AKID) (Parca et al., 2019) (b) *In Vitro* Kinase to-Phosphosite Database (iKiP-DB) which provides data based on a high-throughput *in vitro* kinase assay (Mari et al., 2022), (c) all the kinases of OCIAD1 categorized by Johnson et al. (Mari et al., 2022; Johnson et al., 2023) based on the positional scanning peptide array (PSPA) screening to evaluate the substrate specificity across the kinome, applying a 90 percent cutoff, were also included in the analysis.

The experimentally known protein-protein interactors were compiled from databases including Human Protein Reference Database (HPRD) (Keshava Prasad et al., 2009), Biomolecular Interaction Network Database (BIND) (Bader et al., 2003), BioGRID (Bader et al., 2003; Oughtred et al., 2021), and ConsensusPathDb (Kamburov and Herwig, 2022), CORUM comprehensive resource of mammalian protein complexes (Tsitsiridis et al., 2023), and RegPhos (Huang et al., 2014).

## 3 Results

### 3.1 Mass spectrometry-centric approaches for the identification and analysis of OCIAD1 phosphosites

Although mass spectrometry is widely employed to study PTMs, identifying phosphorylation sites remains a challenge. Over the past decade, the field of phosphoproteomics has rapidly expanded, with an increasing number of phosphoproteome datasets being generated every year. These datasets, derived from a wide range of biological and experimental conditions, often show considerable variation in the reported Class I phosphosites**.**

**FIGURE 1:**
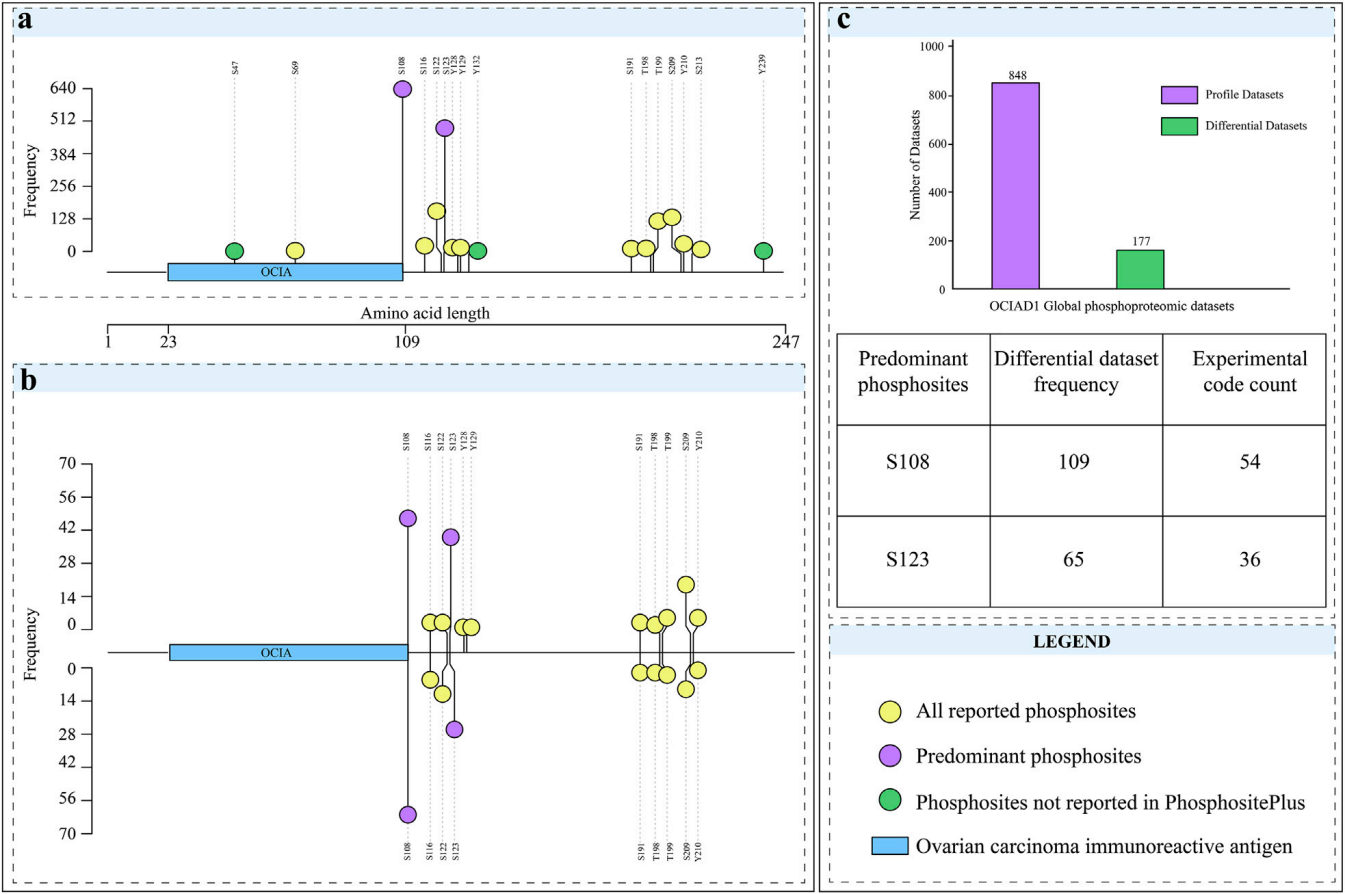
The lollipop plot representing OCIAD1 phosphoproteomic datasets. **(a)** The OCIAD1 phosphosites detected in profile datasets. **(b)** The OCIAD1 phosphosites that are differentially regulated in quantitative differential datasets. **(c)** Number of profiling and differential datasets and the number of datasets in which predominant OCIAD1 phosphosites were differentially regulated.

The screening of around 3,825 published phosphoproteomic research articles resulted in multiple OCIAD1 phosphosites detected in MS-based analysis under distinct experimental conditions. Our analysis compiled 170 research articles with data representation on OCIAD1 phosphosites at the human cellular level, consisting of 849 profiling datasets (biological/experimental conditions that profiled) and 177 quantitative differential datasets (biological/experimental conditions that reported differential expression) (Figure 1). The systematic mapping of profiling data resulted in the gathering of 16 OCIAD1 phosphosites. Notably, the understudied sites Y239 (Bian et al., 2016), S132 (Bian et al., 2016; Chen et al., 2016), and S47 (Bian et al., 2016; Chen et al., 2016; Vacchini et al., 2022), which were not reported previously in PhosphositePlus, were also found. From the differential data across diverse experimental conditions, 11 OCIAD1 phosphosites were found to be differentially regulated, highlighting the dynamic phosphorylation status of OCIAD1 across different biological contexts. The list of profiling and differential datasets of class 1 OCIAD1 phosphosites is given in Supplementary Materials S1, S2.

### 3.2 S108 and S123 are the most frequently phosphorylated sites of OCIAD1

To study OCIAD1 phosphorylation, we prioritized all the identified phosphosites of OCIAD1 based on how frequently they appeared across profiling and differential datasets. Based on our curated phosphoproteomic datasets, the serine residues S108 and S123 are the most detected phosphosites in OCIAD1. They were identified in 640 and 484 profiling datasets and exhibited differential regulation in 109 and 66 differential datasets. They were accounted for approximately 70% of all the datasets in which OCIAD1 phosphosites were reported. Notably, OCIAD1 S108 is located in the OCIA domain of the protein. The multiple-sequence alignment performed using Clustal Omega revealed that the serine residues at positions 108 and 123 are uniquely present in OCIAD1 and are absent in its homolog OCIAD2.

The frequent observation and differential regulation in a large number of datasets across multiple experimental conditions indicate that phosphorylation at these sites is a common and potentially crucial regulatory modification that may influence OCIAD1 functions.

### 3.3 Sequence coverage of OCIAD1 protein

Sequence coverage refers to the proportion of the total observed protein sequence length to the total protein length. It is an important factor in bottom-up proteomics, as it influences the confidence and completeness of protein identification (Shao et al., 2023). Additionally, a higher sequence coverage enables the precise mapping of PTMs, aids in distinguishing protein isoforms, supports quantitative proteomics, and provides insights into the structure of a protein (Shao et al., 2023; Meyer et al., 2011).

**FIGURE 2:**
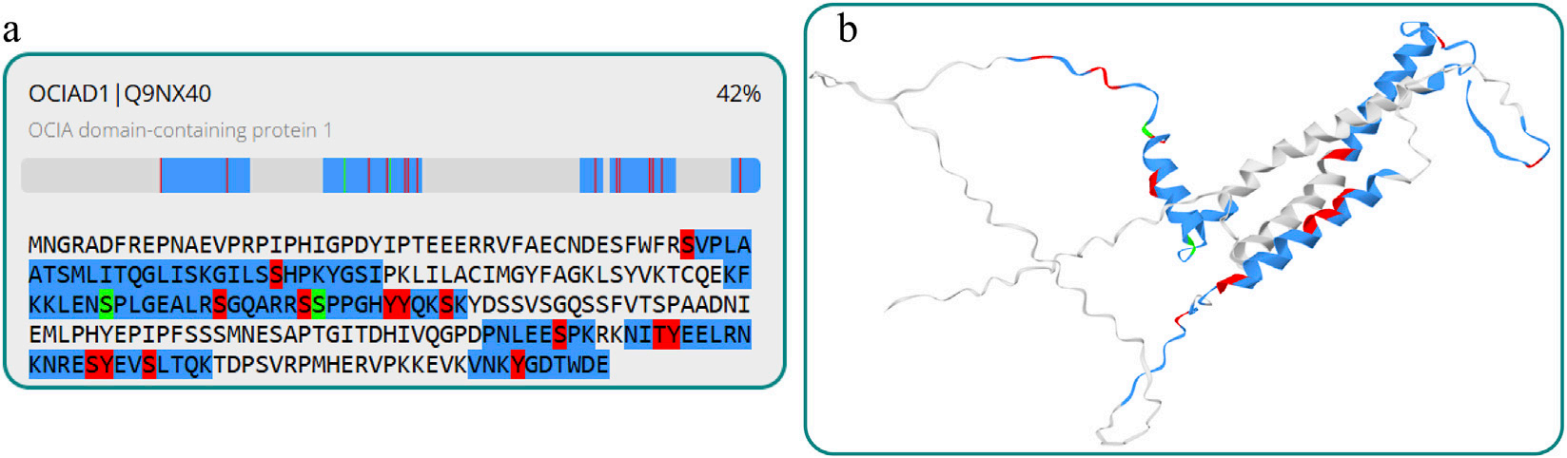
The sequence coverage and the class 1 phosphosites identified from 849 OCIAD1 phosphoproteomic datasets. **(a)** A sequence coverage of 42% was achieved, corresponding to 245 amino acids in OCIAD1. **(b)** The 3D-predicted OCIAD1 model shows peptides identified in profiling data, with red representing the identified OCIAD1 phosphosites, green representing predominant OCIAD1 phosphosites, and blue indicating total sequence coverage.

The analysis of 849 global phosphoproteomic profiling datasets identified 14 unique peptides corresponding to the OCIAD1 protein. These peptides covered 42% of the full 245-amino-acid sequence of OCIAD1. This level of sequence coverage highlights the portion of the protein that is potentially detectable by mass spectrometry-based proteomics. The coverage and corresponding structural representation were visualized using the SCV tool and are presented in Figure 2.

### 3.4 Co-differential phosphorylation networks involving OCIAD1 predominant sites

To identify the regulatory context of OCIAD1 phosphorylation, we examined the phosphosites on co-differentially regulated phosphopeptides belonging to other proteins.

**FIGURE 3:**
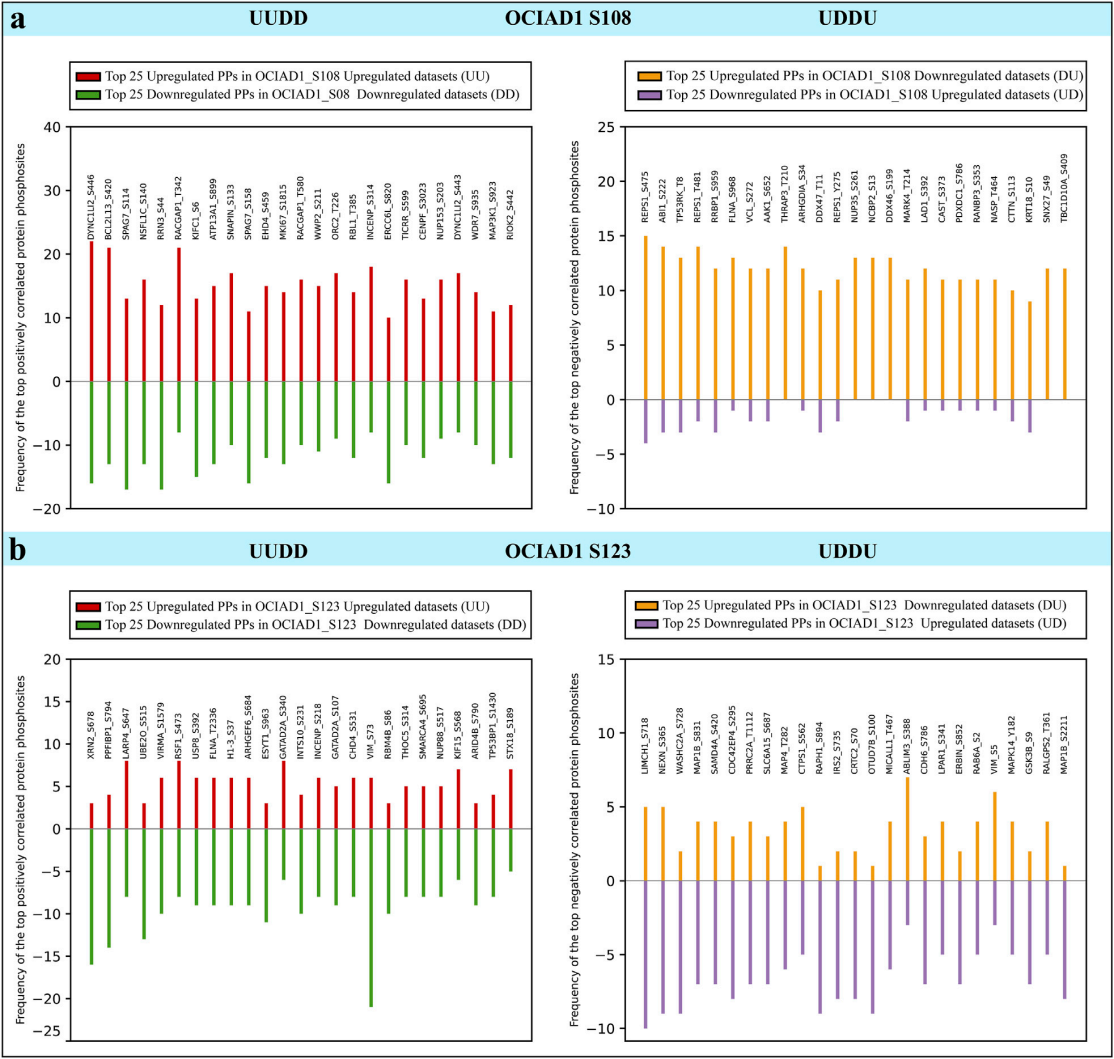
Top 25 protein phosphosites that exhibit co-differential regulation with OCIAD1 predominant sites based on p-value, ratio of co-differential regulation, PMID confidence, and experimental code confidence: **(a)** OCIAD1 S108 and **(b)** OCIAD1 S123.

For this, we initially visualized the differential expression between pairs of OCIAD1 phosphosites using a one-sided Fisher’s exact test (FET), based on a contingency table constructed from the 177 differential datasets. After applying all the filtering strategies, we identified 1,048 and 76 positively and negatively co-differentially regulated protein phosphosites for OCIAD1 S108 and 661 and 207 for S123, respectively. The list of high-confidence protein phosphosites co-differentially regulated with OCIAD1 predominant sites is given in Supplementary Materials S3–S6. The top 25 protein phosphosites that exhibit strong co-differential regulation with predominant OCIAD1 sites are given in Figure 3.

DYNC1LI2_S446 is the top similarly co-differentially regulated phosphosite of OCIAD1 S108 with a differential FET p-value of 3.86299E-08, a ratio of positive co-differential regulation 38:1, co-differentially regulated in 15 studies, and co-differentially regulated in 17 distinct experimental codes (3.86299E-08, 38, 15, 17). REPS1_S475 is the top oppositely co-differentially regulated phosphosite (6.21518E-05, 19:0, 3, 6).

In the case of OCIAD1_S123, the top similarly co-differentially regulated phosphosite is XRN2_S678 (1.84517E-07, 19:1, 8, 10), and the top protein phosphosite that showed opposite co-differential regulation is LIMCH1_S718 (6.53464E-05, 15:1, 8, 10).

Given below are the top protein phosphosites that are co-differentially regulated with OCIAD1 predominant phosphosites along with their FET p-value, confidence ratio for similar or opposite co-differential regulation, confidence of co-differential regulation on individual studies (PMID confidence), and the confidence of co-differential regulation on different stimuli used (experiment codes) (Tables 1, 2). Supplementary Figure S1 presents the cumulative distribution of co-differentially regulated protein phosphosites across distinct experimental conditions.

**TABLE 1:** The top protein phosphosites that exhibit similar co-differential regulation with OCIAD1 predominant sites.

OCIAD1 predominant site	Protein phosphosite	p value	Confidence of similar co-differential regulation	PMID confidence	Experimental code confidence
OCIAD1_S108	DYNC1LI2_S446	3.86299E-08	38:1	15	17
OCIAD1_S108	BCL2L13_S420	4.42136E-06	34:1	12	16
OCIAD1_S123	XRN2_S678	1.84517E-07	19:1	8	10
OCIAD1_S123	PPFIBP1_S794	4.68483E-06	18:1	8	9

**TABLE 2:** The top protein phosphosites that exhibit opposite co-differential regulation with OCIAD1 predominant sites.

OCIAD1 predominant site	Protein phosphosite	p value	Confidence of opposite co-differential regulation	PMID confidence	Experimental code confidence
OCIAD1_S108	REPS1_S475	6.21518E-05	19:1	3	6
OCIAD1_S108	ABI1_S222	0.00174849	17:1	3	5
OCIAD1_S123	LIMCH1_S718	6.53464E-05	15:1	8	10
OCIAD1_S123	NEXN_S365	5.10684E-06	14:1	6	7

### 3.5 The phosphosites in kinases and phosphatases that show co-differential regulation with OCIAD1 predominant phosphosites

Signaling modules are interconnected pathways that regulate various cellular processes. Cross-talk between signaling modules allows for fine-tuning of cellular processes. Although the importance of signaling cross-talk is well recognized, it is challenging to map and visualize these interactions globally. An important process mediating this cross-talk is protein phosphorylation, which is fast, reversible, and highly specific, making it the most efficient means to modulate protein activity and is orchestrated by kinases and phosphatases, which themselves are subject to regulation by phosphorylation (Huttlin et al., 2010; Gopalakrishnan et al., 2025).

**FIGURE 4:**
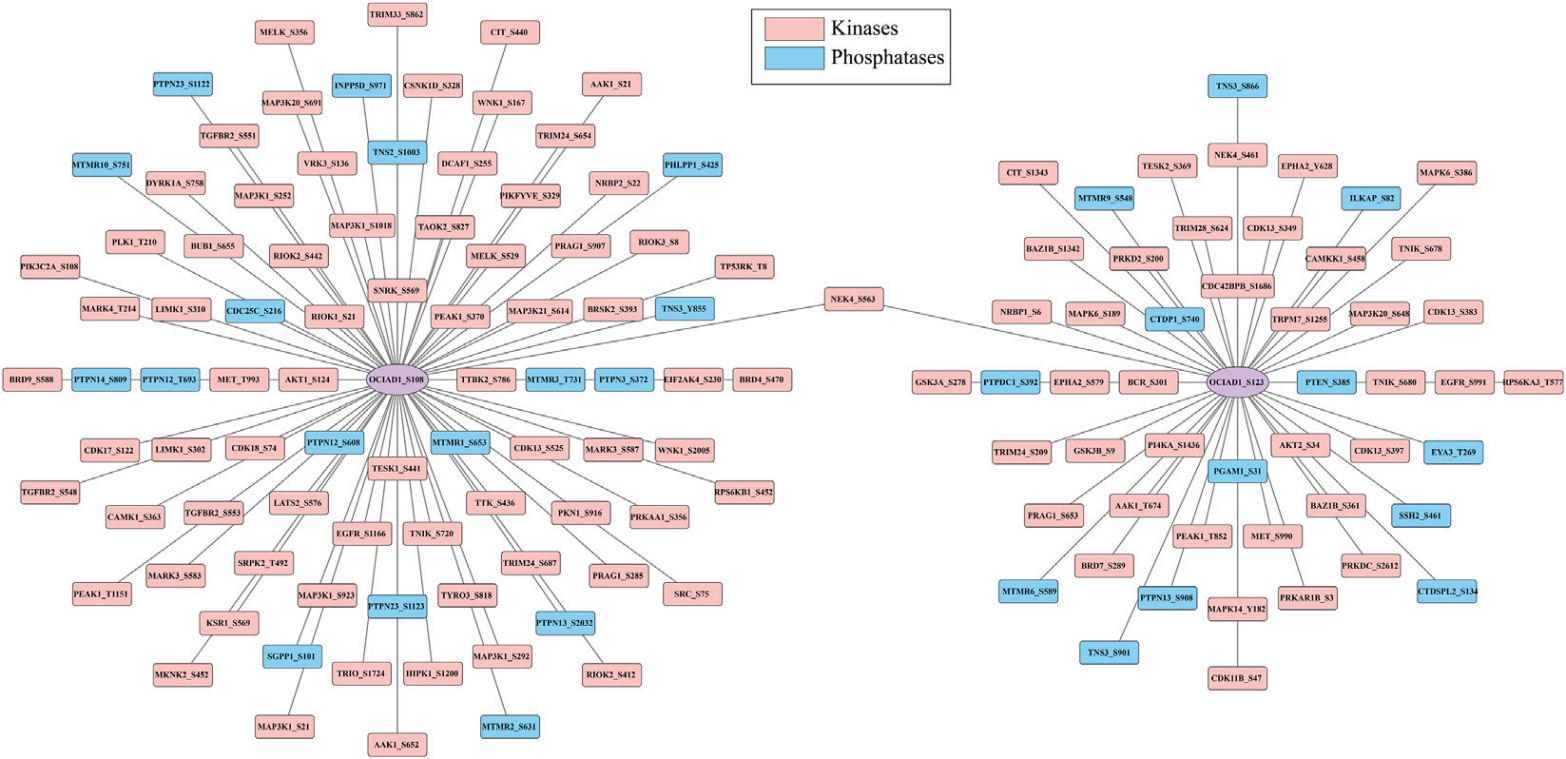
The phosphosites in kinases and phosphatases that exhibit co-differential regulation with the OCIAD1 predominant sites.

To understand the signaling contexts of OCIAD1 phosphorylation, we analysed the similar and opposite co-differentially regulated kinases and phosphatases (Figure 4) (Supplementary Material S7). Among the similarly regulated protein phosphosites of OCIAD1 S108, 68 phosphosites belonged to 54 kinases, and 17 phosphosites were from 15 phosphatases. Three phosphosites coregulated were activity-induced sites of the respective kinases (AKT1 S124, PLK1 T210, and SRPK2 T492), and one of the sites of phosphatases is an inhibition site (CDC25C S216). Phosphosites that were oppositely coregulated belonged to four kinases (4 sites).

For OCIAD1 S123, 25 co-differentially regulated phosphosites were associated with 20 kinases, and 11 phosphosites belonged to 11 phosphatases. Among the coregulated kinase phosphosites, RPS6KA3 T577 was an activity-induced site of its kinase, whereas CAMKK1 S458 functioned as an inhibition site. One of the sites of phosphatases is an activity-induced site (CTDP1 S740). Phosphosites that were oppositely coregulated belonged to 13 kinases and two phosphatases. From the kinases, MAPK14 Y182 is an activity-induced phosphosite, and GSK3B S9 is an inhibition site.

### 3.6 Candidate upstream kinases of OCIAD1

Despite the functional importance of OCIAD1 in carcinogenesis and neurodegenerative disorders, the upstream kinases responsible for their phosphorylation are not yet known. This prompted us to use various approaches for the prediction of upstream kinases, combining high-throughput screening, computational predictions, and literature-derived kinase-substrate specificity data (Supplementary Material S8). First, we queried the iKiP-DB database, which provides data based on a high-throughput *in vitro* kinase assay involving 385 kinases, and identified five kinases, namely EPHA1, EPHA3, LYN, SRMS, and YES1, that may phosphorylate OCIAD1 Y199. In addition, we used computational tools such as NetworKIN and AKID to predict kinase-substrate relationships, resulting in the identification of a total of 82 potential kinases predicted to phosphorylate multiple S, T, and Y phosphosites of OCIAD1. Among these, 14 kinases were consistently predicted to target the predominant phosphorylation sites. Specifically, MAPK13 and MAPK12 were predicted to phosphorylate S108, while CDK2, CDK3, CLK2, PAK3, PRKY, ICK, MAK, MOK, MAPK11, MAPK12, MAPK13, and MAPK14 were predicted to target S123. Since these approaches require individual protein sequences as input, the RefSeq protein sequence of OCIAD1 via their respective APIs was queried.

In addition, we extracted all predicted upstream kinases of OCIAD1 identified by Johnson et *al*. through PSPA analysis based on synthetic peptide screening used to assess kinome-wide substrate specificity, applying a 90th percentile cutoff, thus identifying 195 candidate kinases. Notably, the kinases SRMS and YES1 were consistently found in all three approaches to phosphorylate OCIAD1 Y199 and Y159, underscoring their potential as upstream regulators of OCIAD1.

**FIGURE 5:**
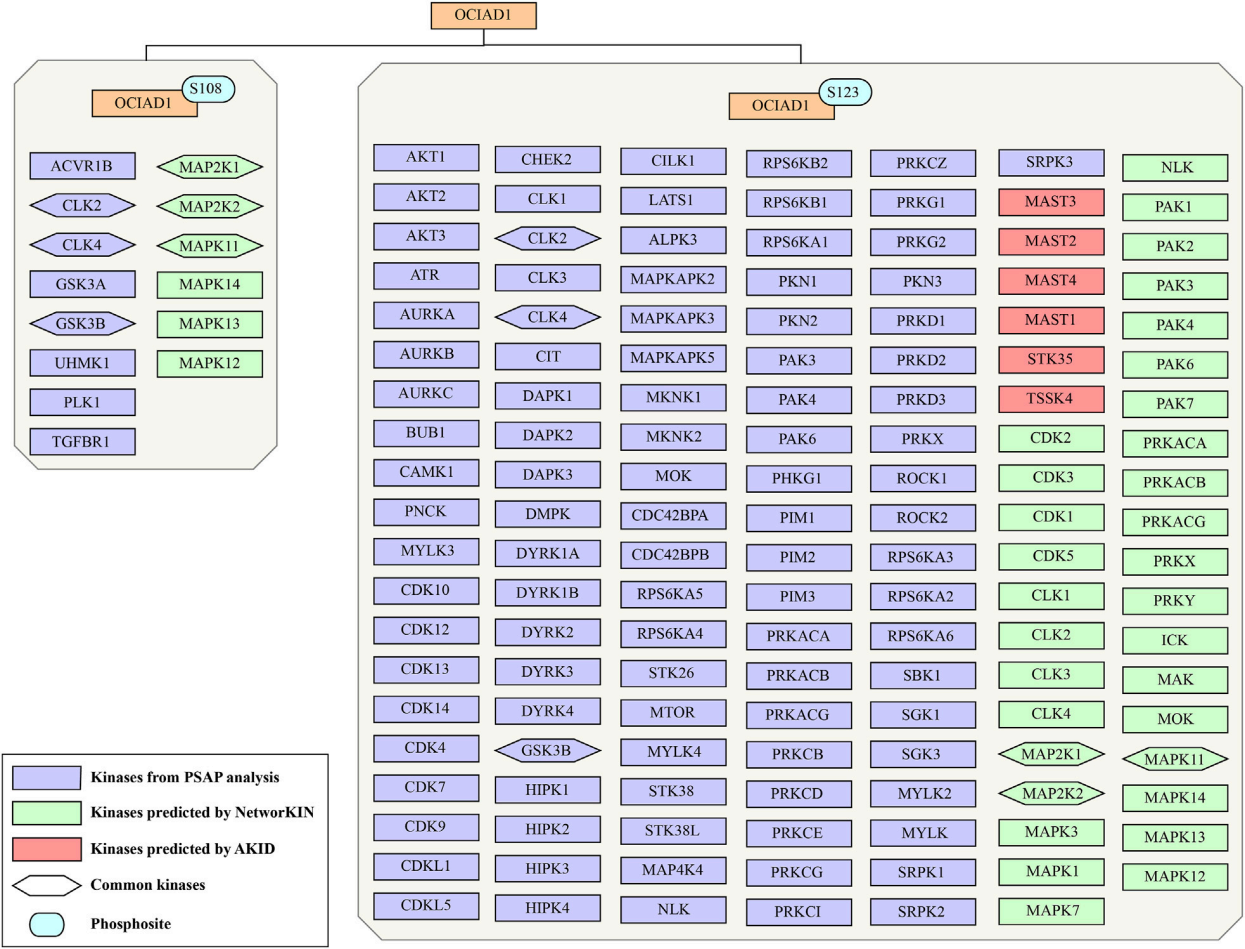
The candidate kinases that may phosphorylate OCIAD1 S108 and S123. The Kinases identified through PSAP analysis are shown in blue, kinases predicted through NetworKIN are shown in green, and kinases predicted using AKID are shown in red.

To add confidence and validate their functional relevance, we examined the co-differential pattern of potential upstream kinases with OCIAD1 predominant sites. Among the potential kinases from the PSPA analysis, phosphosites in five kinases, including PLK1 (T210), CDK13 (S383, S397), PRKD2 (S200), CIT (S1343), and RPS6KA3 (T577), showed strong positive co-differential regulation with OCIAD1 predominant sites, providing confidence in assigning them as upstream kinases. The sites PLK1 (T210) and RPS6KA3 (T577) are enzyme activity-induced sites, indicating that these kinases may be functionally active when OCIAD1 is phosphorylated. The candidate kinases that may phosphorylate the predominant phosphosites of OCIAD1 are given in Figure 5.

### 3.7 The phosphosites in the interactome that are co-differentially regulated with OCIAD1 predominant sites

**FIGURE 6:**
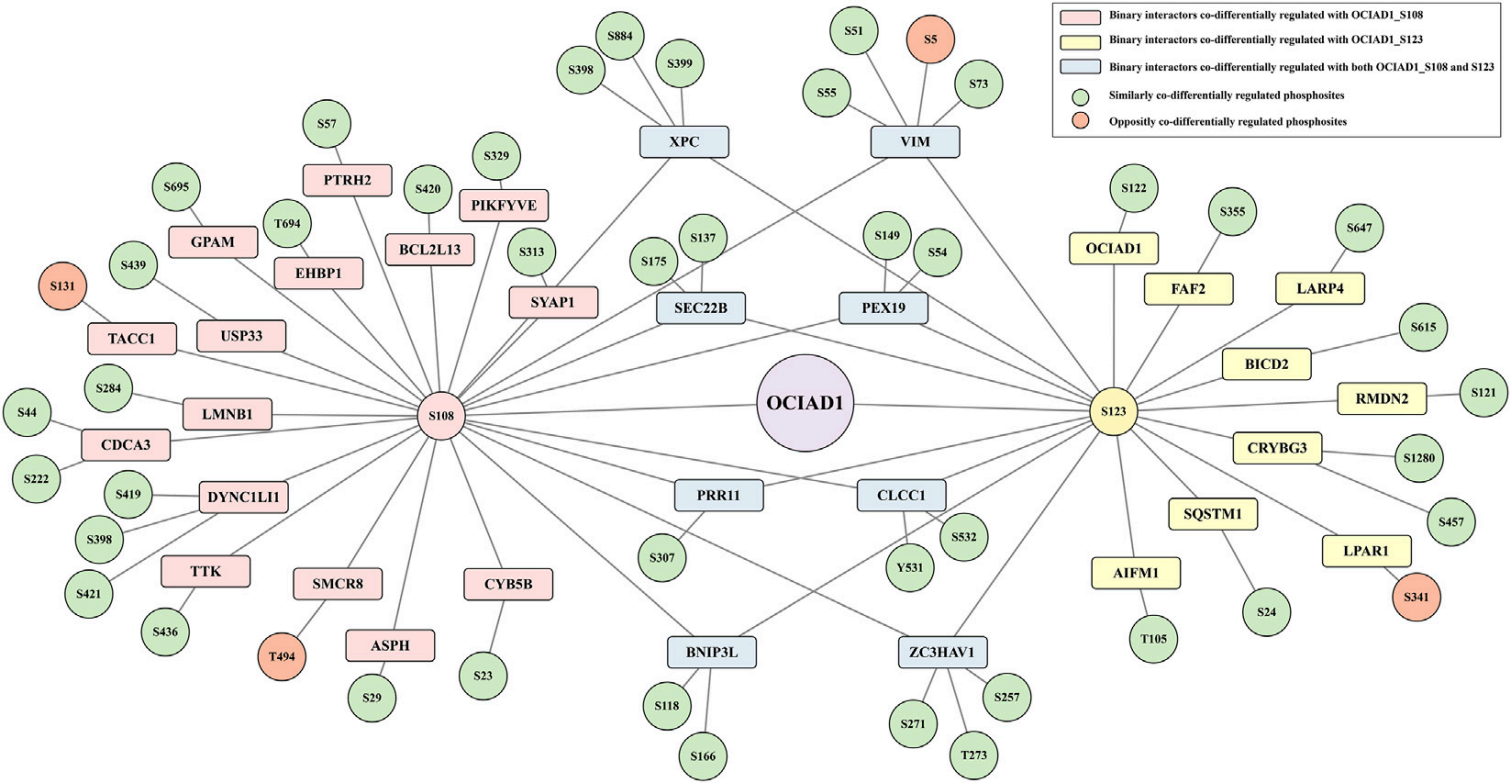
The co-differentially regulated protein phosphosites in the binary interactors of OCIAD1 predominant sites.

We retrieved 230 binary interactors and 31 complexes from various databases, including HPRD, BIND, BioGRID, ConsensusPathDb, CORUM, and RegPhos (SupplementaryMaterial S9). Among these interactors, several phosphosites were co-differentially regulated with OCIAD1 S108 and S123. Specifically, 28 phosphosites belonging to 21 interactors exhibited similar co-regulation with OCIAD1 S108 in the high-confidence phosphosite data list. Likewise, 21 phosphosites from 16 interactors showed similar co-regulation with OCIAD1 S123. Additionally, two phosphosites in two binary interactors showed opposite co-regulation with OCIAD1 S108 and S123. The phosphosites in six of the binary interactors (CLCC1_S532, SEC22B_S175, XPC_S398 and S399, BNIP3L S166, PRR11 S307) were found to be similarly co-regulated with both OCIAD1 S108 and S123 (Figure 6).

### 3.8 The biological process associated with OCIAD1 phosphoregulatory network

To understand the functional aspects of OCIAD1 phosphorylation, a gene enrichment analysis of the proteins harboring phosphosites co-regulated with OCIAD1 predominant sites was performed using Enrichr (Supplementary Material S10) (Kuleshov et al., 2016). The major enriched processes were chromatin remodeling, regulation of DNA-templated transcription, cell cycle, DNA repair, and DNA damage response. In consensus with the known roles of OCIAD1, many proteins were involved in the mitochondrial quality control process, including endocytosis, autophagy, and mitophagy.

**FIGURE 7:**
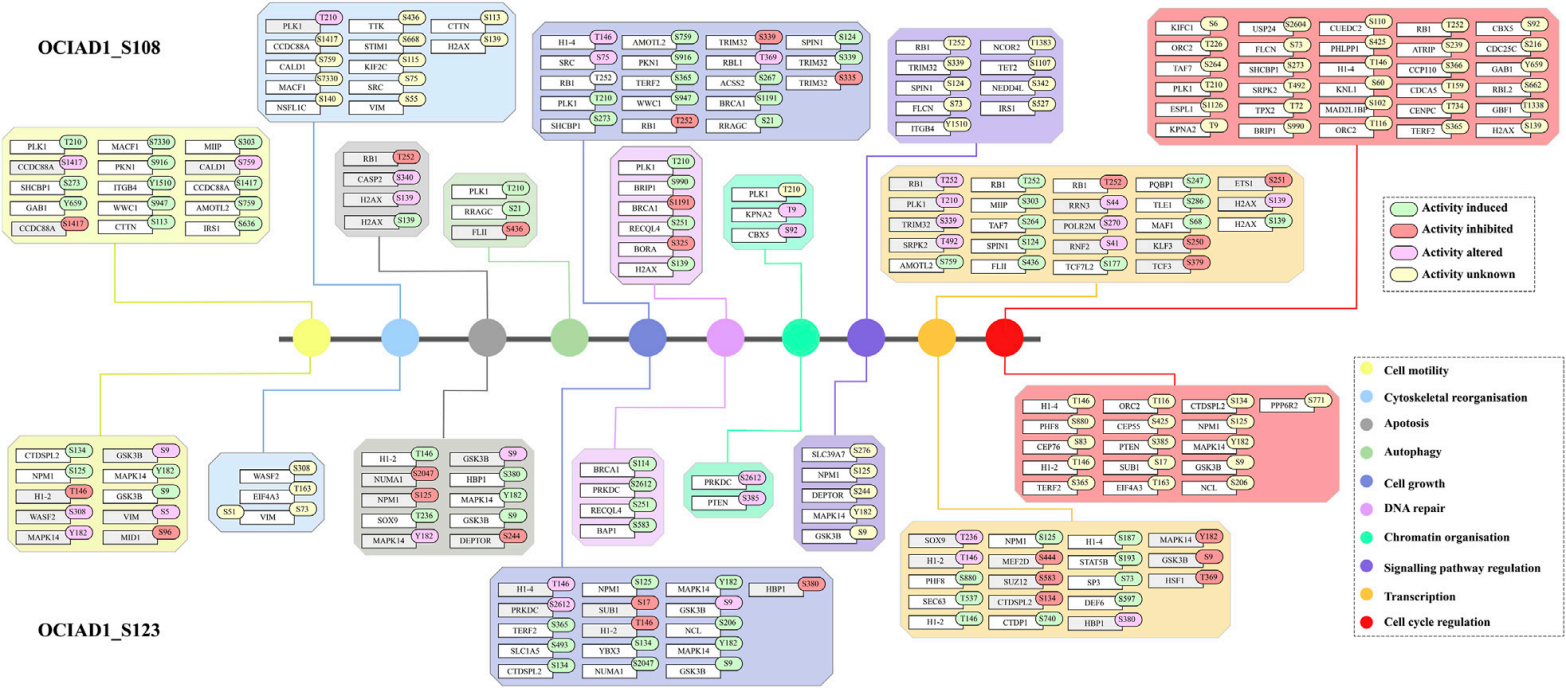
The phosphosite-specific biological processes of the co-differentially regulated protein phosphosites.

Since the study is focused on site-specific phosphoregulation, we decided to investigate the functional annotation of individual phosphosites that were co-regulated with OCIAD1 predominant sites. These sites included both activity-induced and activity-inhibited phosphorylation events, as well as sites exhibiting altered activity (altered function without induction or inhibition). Functional mapping revealed the roles in cell cycle regulation, apoptosis, autophagy, transcription, DNA repair, cell growth, signaling pathway regulation, and cytoskeletal reorganization (Figure 7).

### 3.9 The distribution and differential regulation of OCIAD1 phosphosites across multiple cancers

**FIGURE 8:**
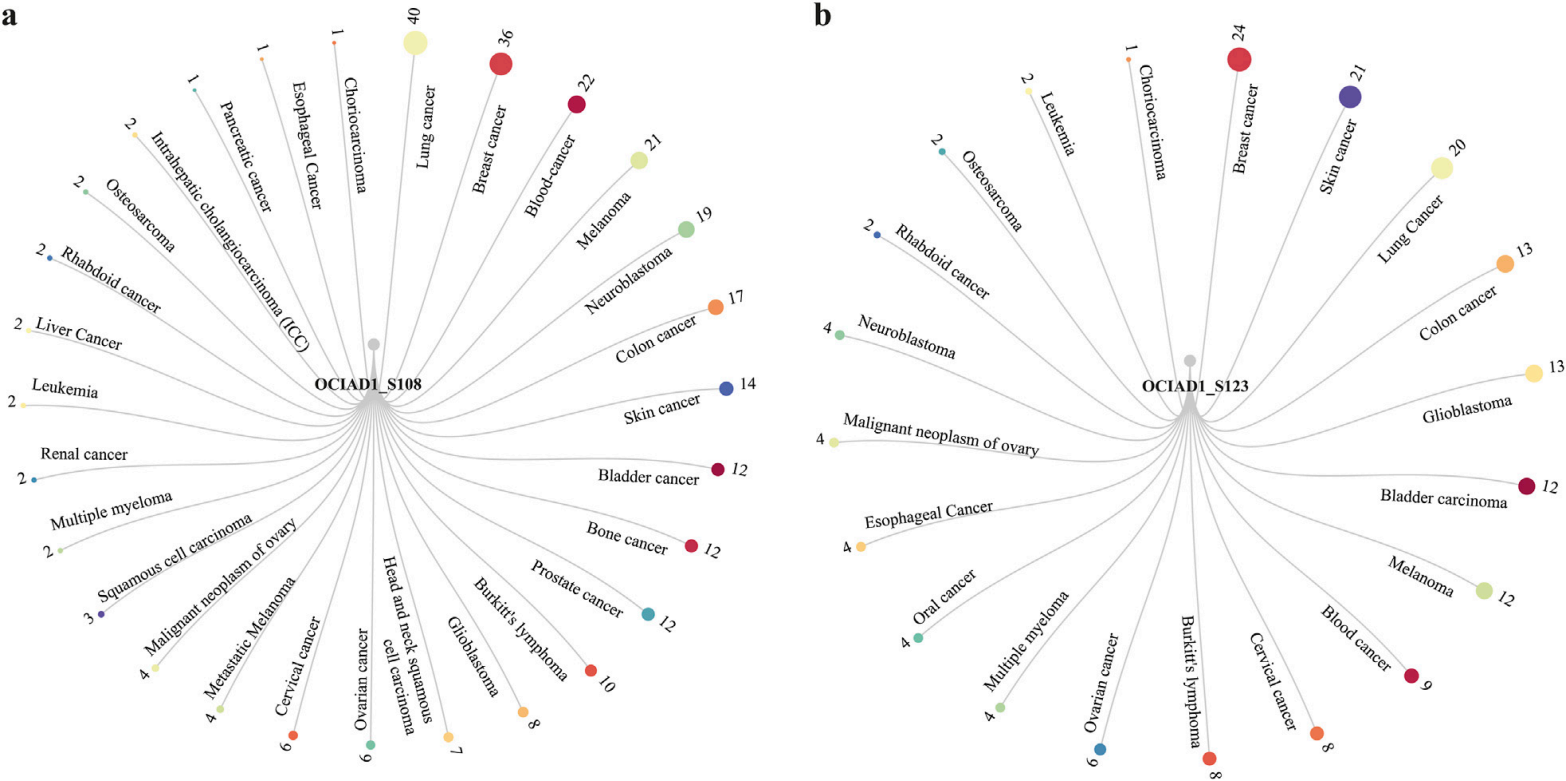
Circular dendrogram representing the distribution of OCIAD1 predominant phosphosites (**(a)** OCIAD1_S108 and **(b)** OCIAD1_S123) identified in various cancer conditions from human profiling data, along with the corresponding number of datasets for each cancer type.

OCIAD1 was reported to be overexpressed and functions as a biomarker in various cancers (Yang et al., 2012). To explore its phosphorylation landscape across various cancers, we analyzed the profiling datasets and found348 datasets containing phosphorylation data for OCIAD1, covering 30 distinct cancers. Across these cancer datasets, nine OCIAD1 phosphosites were identified. The distinct cancers in which OCIAD1 predominant sites are detected, and the number of datasets is given in Figure 8.

To understand the differential expression of OCIAD1 predominant phosphosites in distinct cancer conditions, the human differential datasets were analyzed. OCIAD1 S108 was found to be upregulated in five cancer conditions and downregulated in eight cancer conditions, while phosphorylation at S123 was upregulated in nine cancers and downregulated in seven (Table 3).

**TABLE 3:** Differential expression of OCIAD1 predominant phosphosites (S108 and S123) across cancer types.

Protein	Phosphosite	Expression	Cancers
OCIAD1	S108	Upregulated	Ovarian cancer (Musiani et al., 2014), Lung cancer (Zhang et al., 2021), Melanoma (Schmitt et al., 2019)
OCIAD1	S108	Downregulated	Colorectal cancer (Salovska et al., 2023), Liver cancer (Jing et al., 2021), Neuroblastoma (Pedersen et al., 2021), Breast cancer (Cunningham et al., 2020; Liu et al., 2017; Ruprecht et al., 2017), Glioblastoma (Doll et al., 2017; Narushima et al., 2016), Skin cancer (Galan et al., 2014)
OCIAD1	S123	Upregulated	Skin cancer (Galan et al., 2014), Leukemia (Šalovská et al., 2014), Pancreatic cancer (Paulo et al., 2015), Glioblastoma (Doll et al., 2017), Melanoma (Schmitt et al., 2019), Triple-Negative Breast cancer (Ashrafian et al., 2022)
OCIAD1	S123	Downregulated	Ovarian cancer (Musiani et al., 2014), Lung adenocarcinoma (Hu et al., 2015), Glioblastoma (Narushima et al., 2016), Breast cancer (Ruprecht et al., 2017), Lung cancer (Zhang et al., 2021), Melanoma (Schmitt et al., 2021), Colorectal cancer (Salovska et al., 2023)

### 3.10 Phospho-regulatory network of OCIAD1 and its potential association with mitochondrial quality control and neurodegeneration

The gene enrichment analysis of the co-differentially regulated proteins revealed significant associations with mitochondrial quality control processes, including endocytosis, autophagy, and mitophagy. These proteins had phosphosites that were co-differentially regulated with OCIAD1 predominant sites, suggesting coordinated signaling pathways.

**FIGURE 9:**
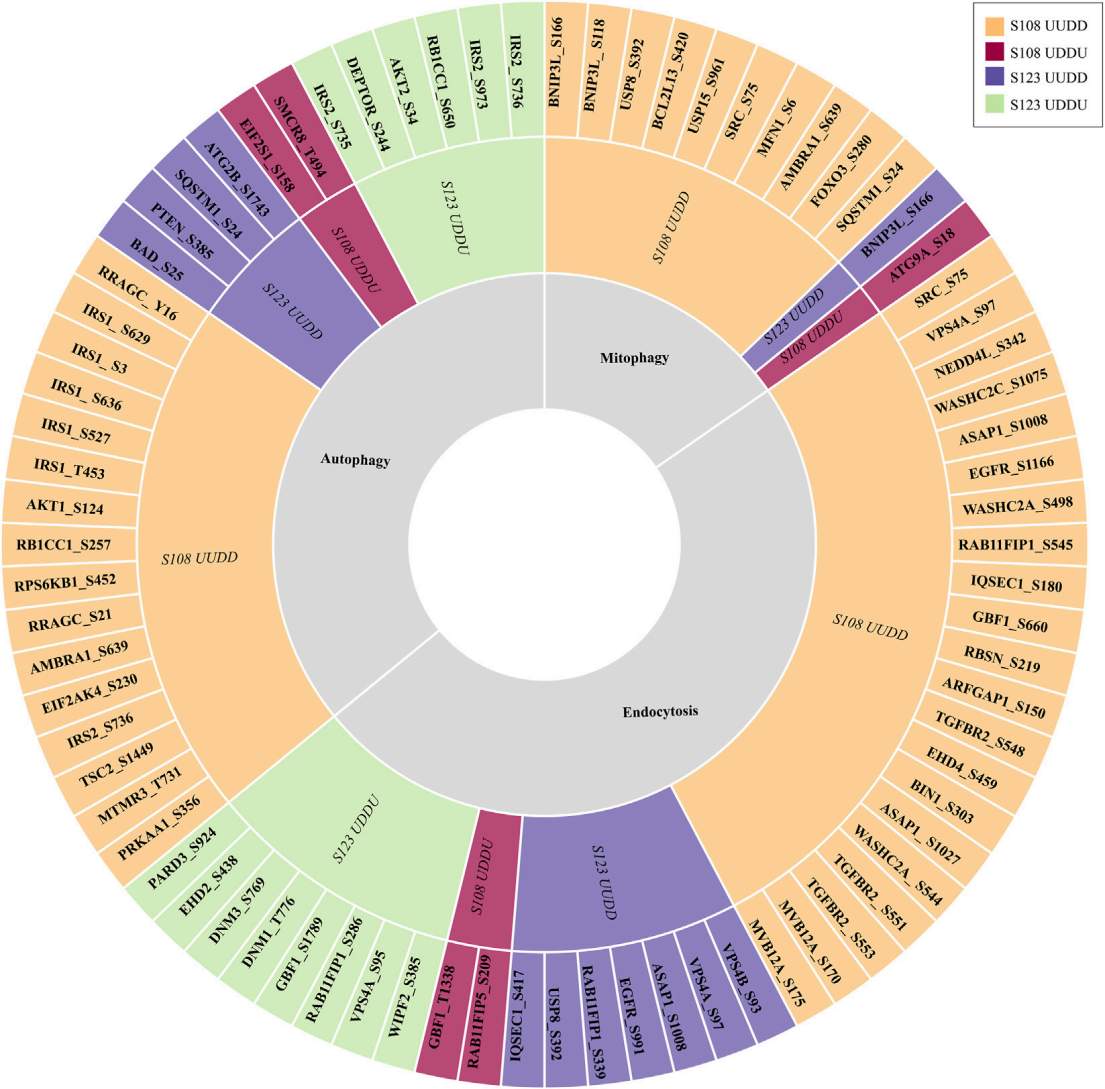
Proteins involved in mitochondrial quality control and their co-differentially regulated phosphosites.

The phosphosites in the key proteins involved in mitophagy and mitochondrial dynamics, such as DYNC1LI2 and REPS1, exhibited strong coregulation with OCIAD1 predominant phosphosites. Additionally, analysis of all five of the co regulated candidate upstream kinases revealed a functional association with mitochondrial regulation. The co-differentially regulated binary interactors were also involved in autophagy and apoptosis. The figure visualizes the proteins involved in mitochondrial quality control and their co-regulated phosphosites (Figure 9).

The GSK3B is a kinase that is prominently involved in the pathology of Alzheimer’s and is known to elevate OCIAD1 levels (Li et al., 2020). Aligning to this, GSK3B was found to be a candidate upstream kinase found from PSPA analysis with a high confidence cutoff and phosphorylating the OCIAD1 predominant sites. Reducing GSK3B levels was found to decrease OCIAD1 levels (Geertsma and Rousseaux, 2020). Notably, the S9 site of GSK3B, an inhibitory phosphosite that reduced its kinase activity, was found to be oppositely coregulated with OCIAD1 S123, meaning that when OCIAD1 phosphorylation is downregulated, phosphorylation at GSK3B S9 is upregulated, and *vice versa*. This implies that GSK3B activity is positively correlated with OCIAD1 phosphorylation. Specifically, increased phosphorylation at GSK3B S9 (which inhibits GSK3B function) coincides with decreased phosphorylation at OCIAD1 S123, suggesting that active GSK3B may directly or indirectly promote OCIAD1 phosphorylation.

Additionally, the disease enrichment analysis of the similarly co-differentially regulated proteins using Enrichr resulted in proteins involved in neurodegeneration pathways, particularly in Alzheimer’s and Parkinson’s, that are upregulated along with OCIAD1 phosphorylation (Supplementary Material S11) (Geertsma and Rousseaux, 2020; Reed et al., 2024).

OCIAD1 has previously been shown to interact with BCL-2, facilitating mitochondrial dysfunction and neuronal injury in Alzheimer’s. Supporting this, our analysis found BCL-2 family member BCL2L13 as a binary interactor of OCIAD1, and its S420 phosphorylation is positively correlated with the OCIAD1 predominant site. BCL2L13 is known to localize to mitochondria and regulate mitophagy, further linking this interaction to mitochondrial quality control (Li et al., 2020). Together, these findings highlight a GSK3B–OCIAD1–BCL2L13 phospho-regulatory relationship that maybe associated with mitochondrial dysfunction and neuronal vulnerability in Alzheimer’s disease. The findings suggest that OCIAD1 may act downstream of GSK3B and interact with mitochondrial apoptotic regulators like BCL2L13, linking OCIAD1 phosphorylation to mitochondrial quality control, warranting further investigation into its possible involvement in neurodegenerative mechanisms.

## 4 Discussions

OCIAD1 is an understudied inner mitochondrial protein belonging to the OCIAD family and is involved in key processes such as mitochondrial quality control, stem cell maintenance, and cell adhesion (Praveen et al., 2020; Shetty et al., 2018; Mukhopadhyay et al., 2003; Calvo et al., 2016; Li et al., 2020). While most of the PTMs in OCIAD2 are ubiquitylation, OCIAD1 is mostly regulated by serine phosphorylation (Praveen et al., 2020). Even though the role of phosphorylation in driving OCIAD1’s function is clear, the functional relevance of any of their phosphosites remains uncharacterized to date. Since protein phosphorylation is responsible for regulating various biological processes, we aimed to analyze OCIAD1 phosphosites and their functional relevance.

Developing highly sensitive, precise MS systems has transformed the investigation of phosphorylation, allowing large-scale phosphoproteomic analysis of unprecedented depth and accuracy. When combined with chromatography-based phosphoprotein and phosphopeptide enrichment methods, MS platforms can detect thousands of phosphorylation sites from a single sample, improving our understanding of dynamic cellular signaling networks and PTMs (Yu and Veenstra, 2021).

The analysis of global phosphoproteomic datasets gathered 11 OCIAD1 phosphosites from different experimental conditions. None of the phosphosite resources provides the differential expression of OCIAD1 phosphosites, making our approach unique. These datasets identified the sites S108 and S123 in OCIAD1 as the predominant phosphosites that were found to be phosphorylated in most of the datasets. Previous studies have shown that OCIAD1 undergoes phosphorylation upon stimulation with lysophosphatidic acid (LPA), specifically at serine residues. In silico analyses identified three putative phosphosites at Ser108, Ser123, and Ser191, and experimental evidence confirmed their phosphorylation. This serine-specific phosphorylation was validated using phospho-serine antibodies, indicating that OCIAD1 is a target of LPA-mediated signaling pathways. These findings suggest that OCIAD1’s functional state is modulated by its phosphorylation status, potentially influencing processes such as drug sensitivity and integrin-mediated cell adhesion (Wang et al., 2010).

Phosphosite co-phosphorylation strongly indicates regulatory coordination within signaling pathways (Jiang et al., 2025). Consistent co-phosphorylation of two or more phosphosites across various conditions implies functional coupling, specificity to a kinase, or participation in a common signaling process. The proteins harboring the top co-differentially regulated protein phosphosites were involved in the mitochondrial quality control processes. Among the top positive regulations, DYNC1LI2 is a microtubule-associated motor protein that plays a crucial role in mitophagy by restoring mitochondrial morphology and function. It prevents excessive mitochondrial fragmentation, improves membrane potential, and enhances mitochondria-lysosome interactions. Additionally, DYNC1LI2 increases PINK1 expression, promoting the removal of damaged mitochondria. It also regulates the localisation of chaperone-mediated autophagy receptor, LAMP2A (Rahman et al., 2022; Hughes et al., 1995). Being a part of exocytosis and mitochondrial dynamics, REPS1 is found to be a potential biomarker of Alzheimer’s and Vascular dementia (Kashatus et al., 2011).

By integrating high-throughput kinase-substrate mapping, computational prediction tools, PSPA analysis, and co-differential phosphoproteomic analyses, we made a comprehensive list of candidate upstream kinases for OCIAD1. The appearance of MAPKs and CDKs, and other stress- and cell-cycle-related kinases (PLK1, PAK3) as regulators of S108 and S123 highlights the possibility that OCIAD1 phosphorylation is involved in cell cycle and stress response pathways (Pellarin et al., 2025; Darling and Cook, 2014; Zhang and Liu, 2002).

The phosphosite-specific functions of the protein phosphosites that are co-regulated with OCIAD1 predominant sites revealed key regulatory networks influenced by phosphorylation events involving OCIAD1 phosphorylation. This analysis revealed a broad spectrum of functions tied to these phosphosites, including cytoskeletal reorganization, transcription, cell cycle regulation, carcinogenesis, DNA repair, autophagy, and apoptosis. Specific alterations in cell growth and motility, as well as modulation of signaling pathways, were also observed. Notably, some phosphosites were linked to both the induction and inhibition of processes such as apoptosis and carcinogenesis.

OCIAD1 has been previously implicated in cancer progression, with its overexpression linked to increased cell migration, invasion, and drug resistance in multiple cancers (Ji et al., 2019; Yang et al., 2012; Sengupta et al., 2008). Our phosphoproteomic analysis significantly extends these observations by providing site-specific phosphorylation profiles across a broad cancer spectrum. The identification of the predominant phosphosites S108 and S123 with differential regulation across multiple cancers emphasizes their potential functional relevance in oncogenic signaling. The observation that the same phosphosite can be upregulated in some cancers and downregulated in others reflects the tumor-type-specific signaling context in which OCIAD1 functions and might arise from distinct upstream kinase activities, cellular stress responses, or metabolic reprogramming. Interestingly, the co-occurrence of OCIAD1 phosphosite dysregulation with mitochondrial and apoptotic pathway components aligns with the emerging view of mitochondria as central hubs in cancer signaling (Ciccarone and Ciriolo, 2024).

## 5 Conclusion

In conclusion, this study utilized global OCIAD1 mass spectrometry datasets to provide the first comprehensive map of OCIAD1 phosphorylation, with the phosphosites S108 and S123 recognised as predominant phosphosites. Our analysis found co-differentially regulated protein phosphosites, revealing broader signaling networks in which OCIAD1 is a part. The study also identified candidate upstream kinases and co-regulated interactors. Our findings suggest that the OCIAD1 phosphoregulatory network maybe associated with oncogenic signaling, and mitochondria-related neurodegeneration indicating its potential utility as a biomarker or therapeutic target in these conditions.

## 6 Limitations and future directions

Despite the comprehensive nature of the study, there are several limitations that need to be addressed while interpreting the findings. This work is based on integrated computational analyses of publicly available datasets. Even though they provide broad coverage of phosphorylation, their heterogeneity in terms of experimental conditions, cell types, and biological systems may introduce variability. While stringent filtering criteria and confidence thresholds were applied to reduce potential bias, complete normalization across datasets remains challenging without access to raw data. In addition, co-differential regulation identified in this study represents statistical associations and does not necessarily indicate direct kinase-substrate relationships or functional interactions. The absence of wet lab experiments is another limitation of the study, as resource constraints prevented the confirmation of the predicted associations.

Future investigations should aim to experimentally validate these phosphosites and their regulatory associations presented in this study. Approaches such as site-directed mutagenesis, targeted studies, and functional assays are important to validate these findings and elucidate OCIAD1’s phosphorylation network. Overall, our study provides a resource for exploring OCIAD1 phosphorylation and lays a foundation for future investigations aimed at uncovering the regulatory significance of OCIAD1 phosphorylation.

## Data Availability

The original contributions presented in the study are included in the article/Supplementary Material, further inquiries can be directed to the corresponding author.

## Supplementary material

SUPPLEMENTARY FIGURE S1 Cumulative distribution of co-differentially regulated protein phosphosites across distinct experimental conditions.
